# Respiratory Syncytial Virus Entry Inhibitors Targeting the F Protein

**DOI:** 10.3390/v5010211

**Published:** 2013-01-16

**Authors:** Zhiwu Sun, Yanbin Pan, Shibo Jiang, Lu Lu

**Affiliations:** 1 Key Laboratory of Medical Molecular Virology of Ministries of Education & Health, Shanghai Medical College and Institute of Medical Microbiology, Fudan University, Shanghai 200032, China; E-Mails: haoyunzhiwu@163.com (Z.S.); shibo.jiang@gmail.com (S.J.); 2 Aris (Nantong) Pharmaceuticals Co. Ltd., Nantong Economic and Technological Area, Jiangsu Province 226006, China; E-Mail: panyanb@yahoo.com

**Keywords:** RSV, viral entry, entry inhibitor, F protein

## Abstract

Human respiratory syncytial virus (RSV) is the main viral cause of respiratory tract infection in infants as well as some elderly and high-risk adults with chronic pulmonary disease and the severely immunocompromised. So far, no specific anti-RSV therapeutics or effective anti-RSV vaccines have been reported. Only one humanized monoclonal antibody, Palivizumab, has been approved for use in high-risk infants to prevent RSV infection. Ribavirin is the only drug licensed for therapy of RSV infection, but its clinical use is limited by its nonspecific anti-RSV activity, toxic effect, and relatively high cost. Therefore, development of novel effective anti-RSV therapeutics is urgently needed. The RSV envelope glycoprotein F plays an important role in RSV fusion with, and entry into, the host cell and, consequently, serves as an attractive target for developing RSV entry inhibitors. This article reviews advances made in studies of the structure and function of the F protein and the development of RSV entry inhibitors targeting it.

## 1. Introduction

Human respiratory syncytial virus (RSV), which was first isolated from chimpanzees with upper respiratory tract illness [1], is a major respiratory pathogen in newborn infants and young children [2]. It can also cause upper and lower respiratory illness in elderly and high-risk adults with underlying chronic pulmonary disease, as well as the severely immunocompromised [3,4]. According to worldwide estimations, RSV was responsible for about 33.8 million lower respiratory tract infections in children younger than 5 years in 2005, with about 3.4 million of them requiring hospitalization. About 66,000–199,000 of these children died of RSV infection, and 99% of these deaths occurred in developing countries [5].

Up to now, no effective vaccine to prevent the spread of RSV has been reported. Palivizumab is the first and only FDA-approved humanized monoclonal antibody (MAb) targeting a virus. It recognizes the “A” antigenic site of RSV F protein to prevent RSV infection in infants and young children at high risk [6,7]. Ribavirin, an indirect inhibitor of RNA transcription, is the only drug licensed for the antiviral treatment of severe RSV infection; however, its effectiveness has not been conclusively established, and its clinical use is limited by its nonspecific anti-RSV activity, toxic effect, and relatively high cost.

Therefore, developing safe and effective antiviral drugs for the treatment and prevention of RSV is urgently needed. This review will focus on the advances made in understanding of the structure and function of the RSV F protein and developing entry inhibitors targeting it.

## 2. Structure and Function of the RSV F Protein

RSV is an enveloped, non-segmented, single-stranded, negative-sense RNA virus belonging to the *Paramyxoviridae* family [8,9]. Its envelope glycoproteins (Env) G and F are responsible for virus attachment and fusion with the target cell membrane. Both glycoproteins contain virus neutralizing epitopes. Because of its higher glycosylation and less conserved sequence, G protein is a less attractive target than F protein for developing anti-RSV vaccines and therapeutics [10,11].

**Figure 1:**
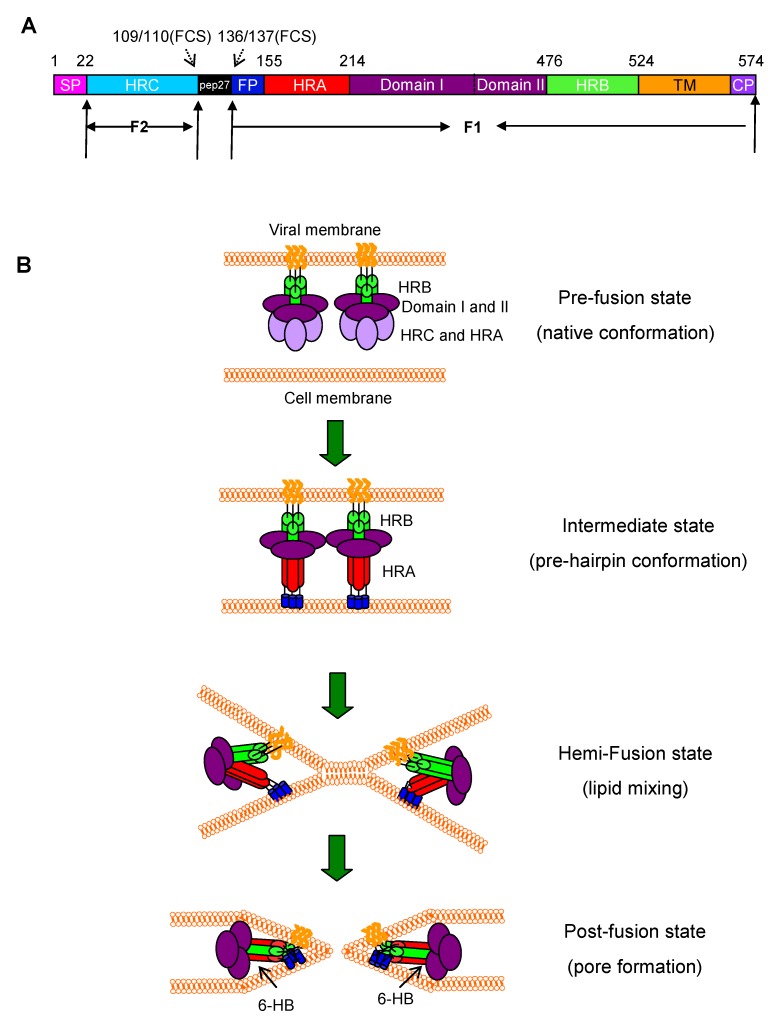
Structure of respiratory syncytial virus (RSV) F protein and RSV fusion/entry processes. (**A**) Schematic representation of RSV F protein. Proteolytic cleavage of the precursor F0 produces the F1 and F2 subunits. Signal peptide (SP), heptad-repeat C (HRC), furin cleavage site (FCS), 27-mer fragment (pep27), putative fusion peptide (FP), domain I and II, heptad-repeat A (HRA), heptad-repeat B (HRB), transmembrane (TM), and cytoplasm (CP) domains are indicated. (**B**) A model of RSV F protein-mediated membrane fusion. In the prefusion state, the FP is buried in the F protein. Once the G protein binds to its receptor(s) on the target cell, the F protein changes conformation into a long HRA helix, at the end of which is FP that inserts into the target cell membrane, and the three HRA domains form a coiled coil trimer (in red). Subsequently, the HRB helices (in green) associate with the HRA trimer to form 6-HB, pulling the cell membrane and viral membrane into close proximity for fusion.

The F protein is a type I transmembrane surface protein, which has an N-terminal cleaved signal peptide and a membrane anchor near the C-terminus [12]. It is synthesized as an inactive 67-kD precursor denoted F0 [13]. In the trans-Golgi complex, the F0 protein is activated proteolytically by furin-like protease at two sites, yielding two disulfide-linked polypeptides, F2 and F1, from the N- and C-terminus, respectively. The 27 amino acid peptide that is released is called ‘pep27’. ‘FCS’ refers to the furin cleavage sites on either side of pep27 [14,15]. The F2 subunit consists of the heptad repeat C (HRC), while the F1 contains the fusion peptide (FP), heptad repeat A (HRA), domain I, domain II, heptad repeat B (HRB), transmembrane domain (TM) and cytoplasmic domain (CP) (Figure 1A) [12,13].

The pre-fusion form of F protein is in a metastable pre-triggered trimer form in the surface of the virus [16]. Its crystal structure has not been solved as yet. However, studies of other paramyxoviruses type I fusion proteins provided a general model for the type I viral fusion proteins. The uncleaved protein folds to a metastable state, which can be activated via a series of conformational changes to a more stable post-fusion state [17]. Recently, Peeples and colleagues[16] produced a pre-triggered soluble F (sF) protein of RSV by deleting the transmembrane and cytoplasmic domains. Consistent with the pre-triggered F protein, the sF protein is in a non-aggregated form with a spherical shape. However, in a low-molarity buffer, the sF aggregates in rosettes, which is the characteristic of the post-triggered form of the sF protein. This pre-triggered sF offers a useful molecular probe to study the attachment and triggering mechanism of RSV F protein [16]. Studies demonstrate that the HRA and HRB can form coiled-coil structures. X-ray crystallographic analysis of the HRA/HRB complexes reveals that three HRAs form a three-stranded coiled-coil bounded by three antiparallel HRBs to form a six-helical bundle core [18]. Last year, two groups have independently solved the atomic structure of the RSV F protein in complete post-fusion conformation through analysis of the version of protein that was removed the fusion peptide, transmembrane domain and cytoplasmic tail [19,20]. The crystallographic analysis of the RSV F post-fusion trimer reveals that the domain I and domain II at the top of the head of F trimer form a crown structure, while HRC and HRA form the base of the head. Besides, HRA extends and forms the trimer coiled coil in the center of the stalk which consists of three HRAs and three HRBs as above described [20].

 It is generally believed that RSV infection begins with the attachment of its glycoprotein (G) to cellular glycosaminoglycans (GAGs), such as heparin sulfate and chondroitin sulfate B [21,22]. However, more and more evidence has shown that RSV infection *in vitro* does not fully depend on G protein-mediated binding to GAGs [23]. Other cellular proteins, such as the intracellular adhesion molecule (ICAM)-1 [24] and nucleolin [25], may also be associated with RSV infection *in vitro*. The recombinant RSV having F protein but lacking G protein could still infect the target cells and induce syncytia *in vitro* with efficiency similar to or lower than that of the wild-type virus, suggesting that, unlike most members of paramyxovirinae, RSV may use F protein to mediate not only membrane fusion, but also viral attachment [23,26].

Like the gp41 of HIV-1, the F protein mediates the fusion of RSV’s envelope with the target cell membrane in a pH-independent manner [18,27]. After the binding of the viral G and F proteins to the receptor(s) on the target cell, the F protein undergoes a series of conformational changes. Its fusion peptide is exposed and inserted into the target cell membrane. Then, its HRB and HRA domains interact to form a stable 6-HB core structure, resulting in membrane apposition. Finally, the fusion pore opens, and viral genetic material enters the target cell (Figure 1B) [18,27]. Alternatively, RSV may enter the host cell through endocytosis mediated by clathrin, since RSV infection can be inhibited by small interfering RNA (siRNA) targeting cytoskeletal dynamics and endosome trafficking genes related to clathrin-mediated endocytosis. If so, the F protein would be needed to cause fusion of the virion membrane with the endocytic vesicle [28].

## 3. Peptide RSV Entry Inhibitors Targeting F Protein

In the early 1990s, Jiang *et al.* [29] and Wild *et al.* [30] identified highly potent anti-HIV peptides derived from the HIV-1 gp41 C-terminal heptad repeat (CHR) region. One of the peptides, T20, was approved in 2003 by the U.S. FDA as the first HIV entry/fusion inhibitor (generic name: Enfuvirtide; brand name: Fuzeon) for treatment of HIV-1infected patients who failed to respond to other antiretroviral drugs. Since RSV has a fusogenic mechanism similar to HIV [31], the approaches developed for discovery of anti-HIV peptides have been applied to identify anti-RSV peptides derived from the HRA and HRB domains in the RSV F protein. Lambert *et al. *[32] designed and synthesized a series of overlapping 35-amino-acid peptides derived from the HRB of RSV F protein. They found that one of the peptides, T-118 (aa 488–522), exhibited highly potent anti-RSV activity, with an EC_50 _value of 0.05 μM (Table 1) [32]. Want *et al.* [33] demonstrated that peptides derived from the HRA of RSV F protein also had inhibitory activity against RSV. For example, the HRA-30a peptide (aa 153–214) displayed strong viral fusion inhibitory activity with an IC_50_ value of 1.68 μM. The same group later constructed three polypeptides containing multiple copies of alternating HRA (aa 156–204) and HRB (aa 485–524), denoted as 5-Helix, HR121 and HR212, and found that they could inhibit RSV infection with an IC_50 _value of 3.36 ± 0.23, 3.74 ± 0.67 and 7.95 ± 1.01 μM, respectively [34]. The larger peptide F478-516 derived from the aa 478–516 in the HRB domain of F protein displayed anti-RSV activity at low micromolar range. If the virus was pretreated with this peptide, it did not interfere with virus infectivity, leading to the conclusion that F478-516 inhibits RSV infection by interacting with the fusion intermediate of the F protein [35]. Similar to the anti-HIV peptides derived from the gp41 CHR region, the shorter peptides derived from the HRB domain of RSV F exhibited lower anti-RSV activity. For instance, the 17-mer peptide C17 (aa 495–511) had no inhibitory activity at the concentration of 100 µM, while the 20-mer peptide C20 (aa 492–511) and the 30-mer peptide C30 (aa 482–511) exhibited anti-RSV activity with IC_50_ values of 14.9 and 6.8 μM, respectively [36] (Table 1). Like the anti-HIV C-peptides, the anti-RSV HRB-peptides also inhibit viral fusion by binding to the HRA trimer and blocking the formation of the 6-HB core [18,27,31].

Although a number of the peptides described above showed potent anti-RSV activity in the *in vitro* tests, none of them was reported in clinical trials, possibly because of such shortcomings as lack of oral availability, high cost of production and relatively low half-life in the circulation. Therefore, development of small-molecule RSV F entry inhibitors is a more desirable goal.

**Table 1:** Inhibitory activity of peptides derived from the RSV F protein heptad repeat B (HRB) region on RSV infection

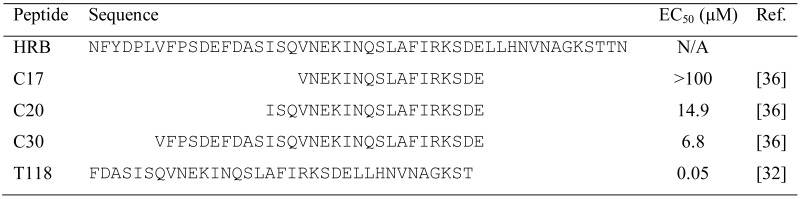

## 4. High-Throughput (HTS) Assays for Screening of RSV Fusion Inhibitors targeting F Protein

To identify anti-RSV compounds, Lundin *et al*. [37] developed a cell-based HTS assay by screening compounds in 384-well plates. HEp-2 cells were seeded in the wells the day prior to the experiment. The test compounds were added to the cells 10 min before addition of the RSV A2 strain. After incubation at 37 °C for 3–4 days, the cells were observed under a microscope for the inhibitory activity of the compounds for RSV-induced cytopathic effect (CPE). Although this cell-based HTS screening assay can be easily adapted in a regular virology laboratory, it is time-consuming and expensive. Besides, the anti-RSV compounds identified using this method may not be the RSV entry inhibitors.

We previously developed the first enzyme-linked immunosorbent assay (ELISA) and first fluoresces-linked immunosorbent assay (FLISA)-based HTS assays [38,39] using an HIV-1 gp41 6-HB-specific monoclonal antibody NC-1 [40] to screen for HIV-1 fusion/entry inhibitors. Using these methods, we have identified a series of small molecule HIV-1 fusion/entry inhibitors, such as NB-2 and NB-64 [41].

**Figure 2:**
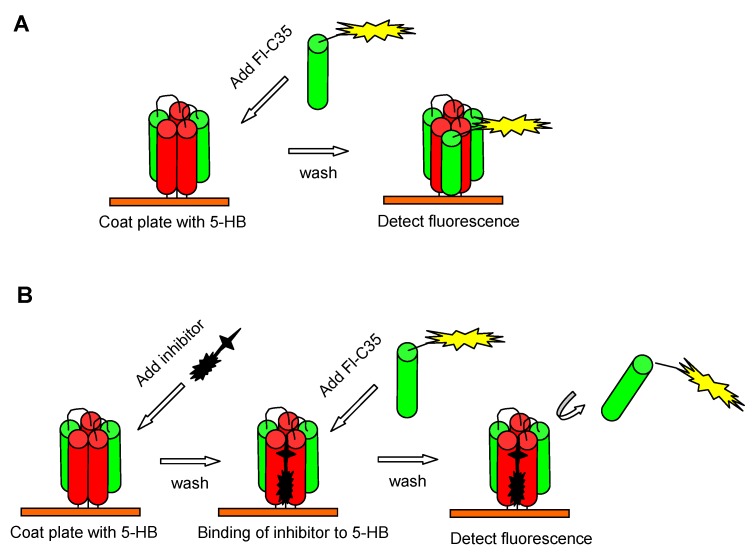
High-throughput (HTS) assays for screening of RSV fusion inhibitors targeting HRA trimer of F protein. (**A**) 6-HB formation by binding FITC-labeled C35 (Fl-C35), as a tracer, to 5-HB that was coated on the well of the culture plate. (**B**) Inhibition of a test compound by competing with Fl-C35 in binding with 5-HB and blocking the 6-HB formation.

Similarly, a noncell-based fluorescence polarization assay was established by Park *et al.* to screen compounds that can inhibit the RSV F protein six-helix bundle (6-HB) formation [36]. They first engineered a five-helix bundle (5-HB) by linking three N-peptides, N57 (aa 126–186), and two C-peptides, C49 (aa 476–524), in an alternating sequence using five short linkers (L) in the following order: N57-L-C49-N57-L-C49-L-N57. As a result, the 5-HB had a large open binding site for the third C-peptide or the potential RSV entry inhibitor targeting the HRA trimer of RSV F. The FITC-labeled C-peptide C35 (FL-C35), which could interact with the 5-HB to form stable 6-HB, acted as a probe to screen peptides or compounds able to compete with FL-C35 for specific binding to the exposed groove on the 5-HBin the fluorescence polarization assay (Figure 2). Using this assay, Park *et al.* have identified several potent anti-RSV peptides derived from the HRB domain of RSV F (Table 1) [36].

## 5. Small-Molecule RSV Entry Inhibitors Targeting F Protein

So far, several small-molecule RSV entry inhibitors targeting F protein have been identified. Compared with the small-molecule HIV entry inhibitors targeting gp41 thus far reported, RSV entry inhibitors exhibit higher antiviral potency *in vitro* (EC_50_ could be up to nano- or picomolar level), suggesting considerable potential for further development as novel anti-RSV therapeutics [42,43]. A number of these compounds have successfully completed preclinical and early clinical studies, but for many reasons, none of them has gone through large-scale human clinical trials.

### CL-387626 and RFI-641

By screening a library of 20,000 compounds using a cell-based assay, the researchers at Wyeth-Ayerst Research identified an active biphenyl analogue, CL387626, which potently inhibited RSV infection with an IC_50_ of 0.05 μM [42]. A single dose (30 mg/kg) of CL-387626 administered intranasally 4 or 5 days prior to virus challenge could significantly protect cotton rats from RSV pulmonary infection [44]. Mechanism studies suggested that CL387626 and its analogues did not inhibit viral attachment, but rather, blocked RSV fusion by targeting F protein. Sequence analysis of the viruses resistant to CL387626 demonstrated that the mutations were located in the highly conserved regions of the F1 subunit [42]. By modification of the chemical structure of CL-387626, an equipotent compound, RFI-614, was discovered [45,46]. RFI-614 is active against RSV type A and B, but it does not inhibit other phylogenetically related parainfluenza viruses, suggesting its specificity against RSV. Similar to CL-387626, RFI-614 targets the RSV fusion step, rather than its attachment [46]. Viruses with G446R mutation in the F protein were resistant to RFI-641 [47,48]. RFI-641 showed more effective protection than CL-387626 against RSV challenge in different animal models, including African green monkeys, BALB/c mice and cotton rats [46,49]. However, neither CL-387626 nor RFI-641 has been further developed by Wyeth-Ayerst Research, which is now a part of Pfizer.

### VP-14637 and MDT-637

Using a high-throughput screening assay, the researchers at ViroPharma, Inc. identified a bis-tetrazole-benzhydrylphenol derivative, VP-14637, as an RSV entry inhibitor [50]. It is one of the most potent anti-RSV compounds with an EC_50_ value of 1.4 nM [51]. The mechanism studies demonstrated that VP-14637 inhibited RSV fusion by interacting with the HRA domain of RSV F protein. VP-14637-induced drug-resistant mutations were located in the HRB domain (mutation D486N, E487D, and F488Y) and the intervening domain between HRA and HRB (mutation K399I and T400A) [51]. The amino acid residue F488 in HRB plays a crucial role in the interaction between the compound and F protein. Replacement of F488 side chain by other noncyclic side chains resulted in viral resistance to VP-14637, indicating that one of phenyl rings of the compound could interact with the phenyl ring of F488 via certain intermolecular reactions. Although VP-14637 went into a Phase I trial, ViroPharma announced the discontinuation of further development of this drug candidate in 2003, partly for strategic reasons. Then, in 2009, VP-14637 was licensed by ViroPharma to MicroDose Therapeutx. It was reformulated as a dry powder for inhalation, being renamed as MDT-637. Preclinical results indicated that MDT-637 could be delivered effectively in both the upper and lower respiratory tract. It is now in a Phase I trial to assess its safety, tolerability and pharmacokinetic profile [52].

### BMS-233675 and BMS-433771

By screening the Bristol-Myers Squibb proprietary chemical deck, the disubstituted benzimidazole derivative BMS-233675 was identified as a potent RSV fusion inhibitor, with EC_50_ and CC_50_ values of 0.34 and 84 μM, respectively [53]. After a series of modifications, BMS-433771, an azabenzimidazole molecule, was found to have better oral availability, higher anti-RSV activity (EC_50_ = 12 nM) and lower cytotoxicity (CC_50_ = >218 μM) [53,54]. As tested in rodents, oral application of BMS-433771 at 5 mg/kg in BALB/c mice and 50 mg/kg in cotton rats resulted in more than one log10 reduction in viral load [55]. BMS-433771 could interact with the hydrophobic pocket formed by HRA trimeric coiled-coils [56], and its drug-resistant mutations, such as F140 and V144, are located in Fusion peptide portion of F protein, confirming that BMS-433771 acts as an RSV entry inhibitor targeting F protein. However, because of the realignment of company priorities, BMS-433771 has not been further evaluated in clinical trials [57].

### JNJ-2408068 and TMC-353121

**Figure 3:**
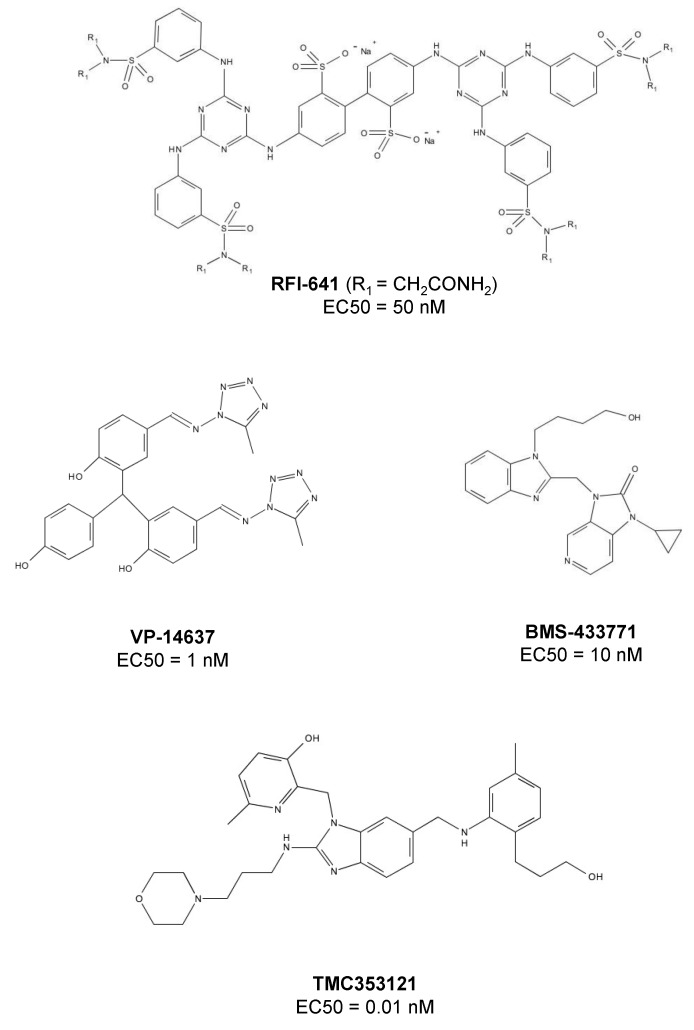
Chemical structures of small-molecule RSV entry inhibitors targeting F protein.

Using an RSV-induced cell fusion assay, researchers at Johnson and Johnson Pharmaceutical Research and Development (Beerse, Belgium) identified a lead compound with anti-RSV activity and then synthesized more than 300 of its analogues. They found that one of the analogues, 2-[[2-[[1-(2-aminoethyl)-4-piperidinyl]amino]-4-methyl-1Hbenzimidazol-1-yl]methyl]-6-methyl-3-pyridinol (JNJ-2408068), exhibited extremely potent anti-RSV activity (EC_50_ = 0.16 nM)[43]. Like VP-14637, JNJ-2408068 also inhibits RSV fusion by binding to ahydrophobic pocket that forms between HAR and HAR during the assembly of 6-HB in the inner core of F protein and interacting simultaneously with both the HRA and HRB domains [18,51]. However, JNJ-2408068 was later found to be unsuitable for further development because of its long tissue retention in rats, dogs and monkeys [58]. Further optimization of JNJ-2408068 resulted in identification a better drug candidate with improved pharmacokinetic profile, TMC-353121, a morpholinopropyl derivative (Figure 3) [59]. TMC-353121 has a better elimination half-life in tissue with picomolar antiviral activity against RSV. *In vitro*, TMC-353121 could inhibit both virus-cell and cell-cell fusion. TMC-353121 was also effective in reducing the viral loads in BALB/c mice after administration of the compound via different routes. As a very promising drug candidate, TMC-353121 is undergoing further development [60].

### BTA-9881

In 2005, researchers at Biota Holdings Ltd. identified an imidazoisoindolone derivative, BTA-9881, as a novel RSV fusion inhibitor [61]. This compound has good oral availability and favorable pharmacokinetics, as proved by AstraZeneca [62]. However, its further development was later stopped because of its unacceptable safety profile. Therefore, its rights were returned to Biota by AstraZeneca. Most recently, however, Biota has described, in a patent, the discovery of a new series of fused imidazopyrazinones as RSV fusion inhibitors with highly potent anti-RSV activity and improved pharmacokinetic properties [63].

## 6. Conclusion and Prospects

It has been more than 60 years since RSV was first isolated. But so far, no effective anti-RSV vaccine or therapeutic modality is available. Palivizumab is the only anti-RSV agent approved for prophylaxis of RSV infection in high-risk populations, but it remains unaffordable for people in developing countries. Ribavirin is the only drug licensed for therapy of RSV infection, but because of its low efficacy and high toxic effect, its clinical use is limited.

The F protein of RSV mediates viral binding and fusion, serving as an important target for development of RSV entry inhibitors. Because of the successful strategies in developing HIV entry inhibitors that target gp41, many researchers in pharmaceutical companies and academic institutions have focused on the identification and development of peptide- and small-molecule-based RSV entry inhibitors targeting F protein. A number of RSV entry inhibitors, such as BMS-433771, RFI-641, VP-14637, and BTA-9881, have gone into clinical trials, but all these trials were discontinued, mainly because of the unfavorable pharmaceutical properties of the compounds. Therefore, the biggest challenge confronting the development of RSV entry inhibitors involves improving the pharmaceutical properties of those active compounds that have already been identified. It is expected that some effective and safe RSV entry inhibitor-based antiviral drugs will be developed in the near future.
